# Predictive value of clinical characteristics on risk and prognosis of synchronous brain metastases in small‐cell lung cancer patients: A population‐based study

**DOI:** 10.1002/cam4.4978

**Published:** 2022-07-13

**Authors:** Gang Zhou, Zhiyuan Zhang, Peng Yu, Ruixuan Geng, Guige Wang, Wenbin Ma, Jun Gao, Zhibo Zheng, Yongning Li

**Affiliations:** ^1^ Department of Neurosurgery, Peking Union Medical College Hospital Chinese Academy of Medical Sciences Beijing China; ^2^ Department of International Medical Services, Peking Union Medical College Hospital Chinese Academy of Medical Sciences Beijing China; ^3^ Department of Thoracic Surgery, Peking Union Medical College Hospital Chinese Academy of Medical Sciences Beijing China

**Keywords:** prognosis, risk factors, SEER, small cell lung cancer, synchronous brain metastases

## Abstract

**Background:**

Patients with small‐cell lung cancer (SCLC) have a high incidence of synchronous brain metastases (SBM) and a poor prognosis, which causes a heavy burden of morbidity and mortality. A better understanding of the demographic and tumor‐specific characteristics of these patients is critical to guiding clinical practice. The purpose of this study was to investigate the predictive and prognostic value of the clinical characteristics of SCLC patients with SBM at initial diagnosis.

**Methods:**

This is a retrospective study based on the data in the latest Surveillance, Epidemiology, and End Results (SEER) version which was released in 2021 for patients diagnosed with SCLC in the presence or absence of SBM from 2010 to 2018. Multivariable logistic regression was performed to identify predictors of the presence of SBM at the initial diagnosis. Kaplan–Meier curves and multivariable Cox regression models were built to compare the prognosis of patients with different clinical characteristics and treatments.

**Results:**

A total of 33,169 SCLC patients were enrolled in this study, including 5711 (17.2%) patients with SBM and 27,458 (82.8%) patients without SBM. Patients who are black(HR = 1.313, 95% CI = 1.167–1.478, *p* < 0.001), higher T stage (T2, HR = 1.193, 95%CI = 1.065–1.348, *p* = 0.005; T3, HR = 1.169, 95%CI = 1.029–1.327, *p* = 0.016; T4, HR = 1.259, 95%CI = 1.117–1.418, *p* < 0.001), lung metastases (HR = 1.434, 95%CI = 1.294–1.588, *p* < 0.001) and bone metastases (HR = 1.311, 95% CI = 1.205–1.426, *p* < 0.001) had greater odds of SBM at initial diagnosis. The median overall survival (OS) for SCLC patients with SBM was 5.0 months. Multivariable Cox regression revealed that age ≥ 65 (HR = 1.164, 95% CI = 1.086–1.247, *p* < 0.025), singled (HR = 1.095, 95% CI = 1.020–1.174, *p* = 0.012), higher T stage (T3, HR = 1.265, 95% CI = 1.123–1.425, *p* < 0.001; T4, HR = 1.192, 95% CI = 1.066–1.332, *p* = 0.002), higher N stage (N2, HR = 1.347, 95%CI = 1.214–1.494, *p* < 0.001; N3, HR = 1.452, 95%CI = 1.292–1.632, *p* < 0.001), liver metastases (HR = 1.415, 95%CI = 1.306–1.533, *p* < 0.001), and bone metastases (adjusted HR = 1.126, 95%CI = 1.039–1.221, *p* = 0.004). Analysis of treatment regimens showed that patients who received combinational treatment exhibited longer OS than chemotherapy or radiotherapy alone, and surgery combined with chemotherapy and radiotherapy exhibited the longest OS.

**Conclusions:**

In this study, we identified risk factors for SBM in SCLC patients and prognostic indicators among this patient population. We also found that patients who received different therapeutic strategies exhibited significant difference on OS, which will provide evidence‐based support for treatment options.

## Graphical abstract

Our study aims to investigate the predictive and prognostic value of the clinical characteristics of small‐cell lung cancer (SCLC) patients with synchronous brain metastases (SBM) at initial diagnosis. The result shows that patients with higher T stage, bone metastases, and lung metastases have greater odds of SBM at initial diagnosis. Age ≥ 65, higher T stage (T3/T4), higher N stage (N2/N3), synchronous liver or bone metastases are confirmed to be predictors of poor prognosis of SCLC patients with SBM. Analysis of therapeutic strategies showed that the combination treatment of chemotherapy and radiotherapy benefits patients more than chemotherapy or radiotherapy alone.

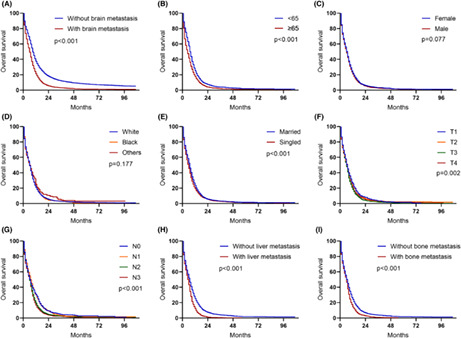

## 1 INTRODUCTION

SCLC is a kind of highly aggressive neoplasm that comprises approximately 14% of all newly diagnosed lung cancers worldwide per year.^1^ Currently, the systemic treatment for SCLC patients follows a comprehensive pattern by integrating surgery, chemotherapy, radiotherapy, and immunotherapy. However, there has been no effective therapeutic breakthrough established in the past few decades. The overall 5‐year survival rate of SCLC patients remains less than 7%, and most patients only survive for 1 year or shorter.^1^ SCLC is characterized by a rapid doubling time, early development to extensive‐stage, and widespread metastases at the time of diagnosis. One of the most frequent metastatic sites is the brain. Compared to other primary malignancies, SCLC has been reported to have the highest rate of SBM at the initial diagnosis.^2^ Previous studies have demonstrated that the rate of synchronous brain metastases of SCLC patients at initial diagnosis is 10%–18% detected by computed tomography (CT) and up to 24% of patients by magnetic resonance imaging (MRI). At the time of death, the rate of brain metastases in SCLC patients is even much higher than 50%.^3^ The high susceptibility of brain metastases indicates the necessity of prophylactic cranial irradiation (PCI) to prevent intracranial metastases in patients with a predisposition. However, studies revealing the clinical characteristics of SCLC patients that are prone to developing brain metastases are limited. Brain metastases generally lead to poor prognosis and cause a significant burden of morbidity and mortality in SCLC patients. Although PCI can improve overall survival (OS) and prevent intracranial metastases in limited‐stage SCLC (LS‐SCLC) patients, the effect on extensive‐stage SCLC patients is debated.^4^ Immediate whole‐brain radiotherapy (WBRT), WBRT with a focal radiation boost, stereotactic radiosurgery (SRS), chemotherapy, and supportive therapy are the main options for SCLC with SBM, but the prognosis of patients in response to the treatments varies significantly. The current evidence does not explain the discrepancies of therapeutic responses and there are still a lot of unsettled questions about individualized treatment.^5^ Therefore, population‐based estimates of the probability and prognosis of newly diagnosed SCLC with SBM are critical to clinical decision‐making.

The Surveillance, Epidemiology, and End Results (SEER) database is a national cancer registry that covers approximately one‐third of the United States population and collects data on cancer incidence, treatment, and survival. In order to provide evidence for clinical practice, we collected data from the SEER database in this study to characterize the demographic and tumor‐specific factors associated with increased propensity of brain metastases in SCLC patients at a population‐based level. We also examined factors related to the prognosis of SCLC patients with SBM and compared the OS of patients receiving different treatment regimes.

## 2 METHODS

Data of SCLC patients registered in the SEER program between 2010 and 2018 were collected and evaluated in this study. Patients missing the pivotal information were excluded from the cohort (e.g., metastasis information, and follow‐up information). The clinical characteristics were classified into three parts: Demographic factors, tumor‐specific factors, and treatment regimens. Demographic information included age at diagnosis, gender, race, and marital status. According to the age distribution, patients were divided into the younger group (less than 65 years) and the elder group (65 years or older). Marital status was divided into married and single. The latter contained patients who were unmarried, divorced, and bereft. The race was divided into white people, black people, and others. In the latest SEER database, insurance status cannot be obtained anymore. Tumor‐specific factors contained the tumor location, tumor laterality, number of primary tumor sites, T stage (tumor size), N stage, and synchronous tumor metastasis. The tumor location was stratified into the upper lobe, the middle lobe, the lower lobe, the overlapping lesion, and other sites. Tumor laterality was stratified into left, right, both, and unspecific. Synchronous tumor metastases include lung metastases, bone metastases, and liver metastases. The treatment regimen included chemotherapy, radiotherapy, and surgery which refers to the resection of primary pulmonary tumors. The type of radiotherapy (primary tumor, PCI, treatment of metastases) cannot be obtained from SEER currently. All these variables were analyzed by the chi‐square test in Table 1. We also calculated the cancer specific survival of SCLC patients. To better characterize the risk factors of SBM in SCLC patients, we performed multivariable logistic regression with these factors. Overall survival (OS) was calculated from diagnosis to death for any cause. The Kaplan–Meier method was used to evaluate OS differences of patients. We further utilized a log‐rank test to compare the survival curves of SCLC patients with SBM, which were grouped by certain clinical characteristics. In addition, both univariate and multivariable Cox proportional hazards models were built to determine which characteristics were independently associated with patients' survival. *p* < 0.05 was considered statistically significant.

**TABLE 1:** Clinical characteristics and cancer‐specific survival (CSS) of SCLC patients with and without brain metastases

Characteristics	With brain metastases *N* = 5711 (17.2%)	Without brain metastases *N* = 27,458 (82.8%)	*P* value
Age			<0.001
<65	2662 (46.6)	10,379 (37.8)	
≥65	3049 (53.4)	17,079 (62.2)	
Gender			<0.001
Male	3024 (53.0)	13,642 (49.7)	
Female	2687 (47.0)	13,816 (50.3)	
Race			<0.001
White	4808 (84.2)	23,762 (86.5)	
Black	611 (10.7)	2432 (8.9)	
Other	292 (5.1)	1264 (4.6)	
Marital status			<0.001
Married	2832 (49.6)	12,926 (47.1)	
Singled	2673 (46.8)	13,284 (48.4)	
Unknown	206 (3.6)	1248 (4.5)	
Tumor location			0.849
Upper lobe	3040 (53.2)	14,482 (52.7)	
Middle lobe	248 (4.3)	1228 (4.5)	
Lower lobe	1329 (23.3)	6337 (23.1)	
Overlapping lesion	86 (1.5)	446 (1.6)	
Other sites	1008 (17.7)	4965 (18.1)	
Laterality			<0.001
Left	2321 (40.6)	10,940 (39.8)	
Right	3026 (53.0)	15,123 (55.1)	
Both	346 (6.1)	1299 (4.7)	
Unspecific	18 (0.3)	96 (0.3)	
Number of primary sites			<0.001
1	3260 (57.1)	16,535 (60.2)	
≥2	1352 (23.7)	5792 (21.1)	
Unspecific	1099 (19.2)	5131 (18.7)	
T‐stage			<0.001
T1	610 (10.7)	3713 (13.5)	
T2	1221 (21.4)	6133 (22.3)	
T3	1092 (19.1)	5170 (18.8)	
T4	1954 (34.2)	8576 (31.2)	
Unspecific	834 (14.6)	3866 (14.1)	
N‐stage			<0.001
N0	809 (14.2)	4117 (15.0)	
N1	469 (8.2)	2052 (7.5)	
N2	2814 (49.3)	14,306 (52.1)	
N3	1302 (22.8)	5765 (21.0)	
Unspecific	317 (5.6)	1218 (4.4)	
Liver metastases			0.392
No	3883 (68.0)	18,828 (68.6)	
Yes	1828 (32.0)	8630 (31.4)	
Lung metastases			<0.001
No	4670 (81.8)	23,968 (87.3)	
Yes	1041 (18.2)	3490 (12.7)	
Bone metastases			<0.001
No	4064 (71.2)	21,240 (77.4)	
Yes	1647 (28.8)	6218 (22.6)	
Chemotherapy			0.325
No	1739 (30.5)	8181 (29.8)	
Yes	3972 (69.5)	19,277 (70.2)	
Radiotherapy			<0.001
No	1581 (27.7)	15,358 (55.9)	
Yes	4130 (72.3)	12,100 (44.1)	
Surgery			<0.001
No	5650 (98.9)	26,519 (96.6)	
Yes	49 (0.9)	860 (3.1)	
Unspecific	12 (0.2)	79 (0.3)	
Survival			<0.001
1‐year cancer specific survival	19.8%	34.3%	
3‐year cancer specific survival	3.0%	11.9%	
Median survival time (m)	5	8	

We also compared the effects of different treatments on patients' survival in SCLC patients with SBM. Because surgery only is not recommended as an option in SCLC patients with SBM in the guideline, and patients who received surgery, surgery combined with chemotherapy, surgery combined with radiotherapy just took a quite small proportion (15 patients, 0.26%), we removed this part of patients and divided the treatment regimens into five groups: Chemotherapy, radiotherapy, chemotherapy combined with radiotherapy, surgery combined with chemotherapy and radiotherapy, and no treatment at all. The Kaplan–Meier curve was used to compare the OS of different groups.

## 3 RESULTS

### 3.1 Clinical characteristics and predictors of SBM in SCLC patients

**FIGURE 1:**
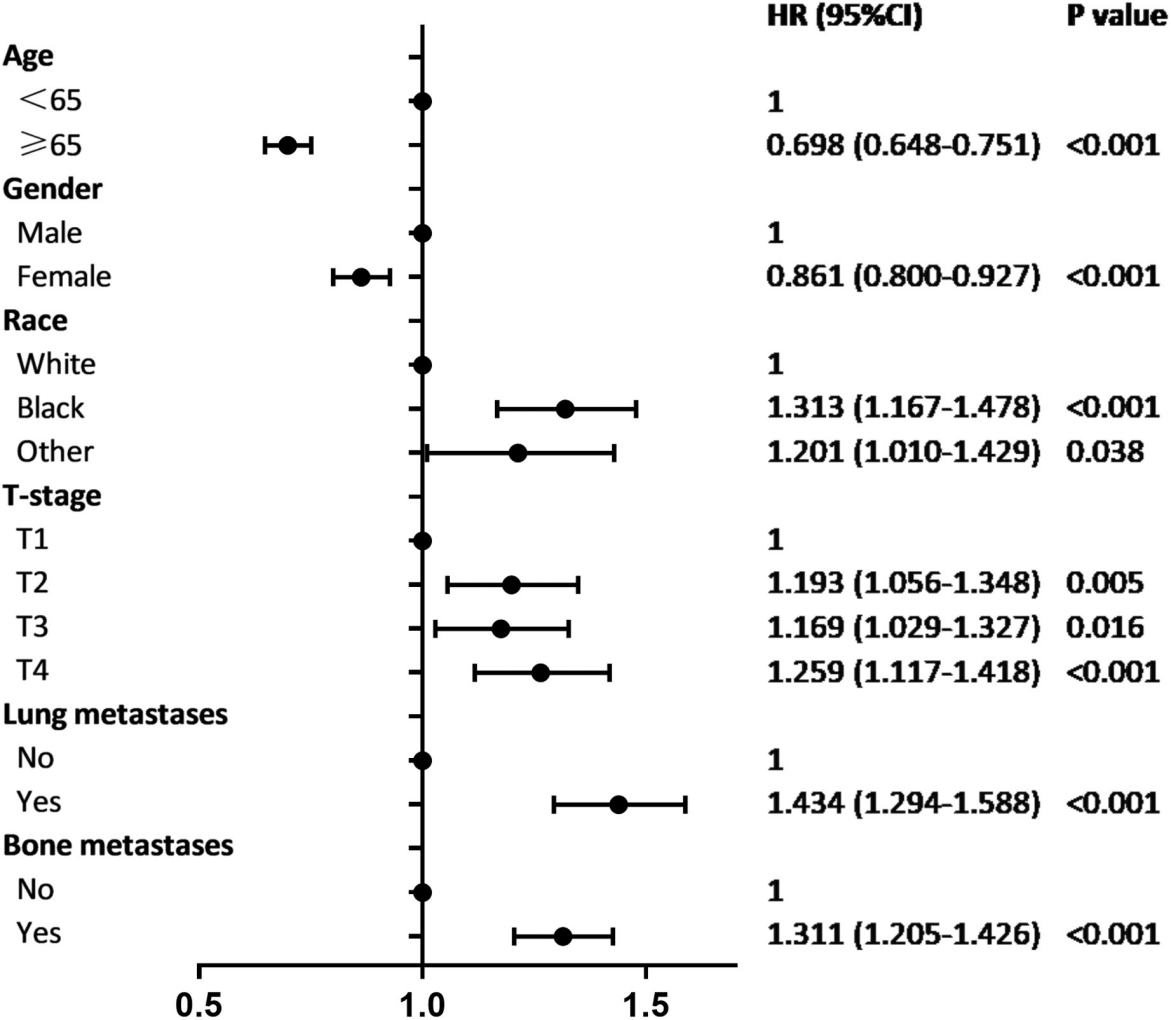
Multivariable logistic regression analysis for the risk of SBM at the initial diagnosis of SCLC patients.

After the patient selection process, 33,169 eligible SCLC patients were enrolled in this study and 5711 (17.2%) of them developed SBM at the initial diagnosis. Table 1 shows the clinical characteristics of all patients in the cohort. In the demographic information part, the proportion of young patients (age < 65) in the SBM group was higher than the non‐SBM group. No significant gender predominance was found in all SCLC patients, but males were more likely to be present with SBM. In terms of race distribution, white people accounted for the majority in the cohort, but black people had a higher incidence of SBM. Marital status also differed between the SBM group and the non‐SBM group. In tumor characteristics part, SCLC patients with SBM had lower rate to be located on right lung, higher rates to have more than one site, higher rate to be T3 and T4 stage, higher rate to be N1 and N3 stage, higher rates of bone and lung metastases. However, the tumor locations in different lobes and liver metastases did not show significant differences between the two groups. We also performed multivariate logistic regression to identify which factors could be predictors of SBM among patients with SCLC in Figure 1. The result shows elder patients and female were protective factors of having SBM at diagnosis. Whereas, black patients, higher T stage, lung metastases, and bone metastases were risk predictors of having SBM at diagnosis.

### 3.2 Prognosis and survival analysis of SCLC patients with SBM

**FIGURE 2:**
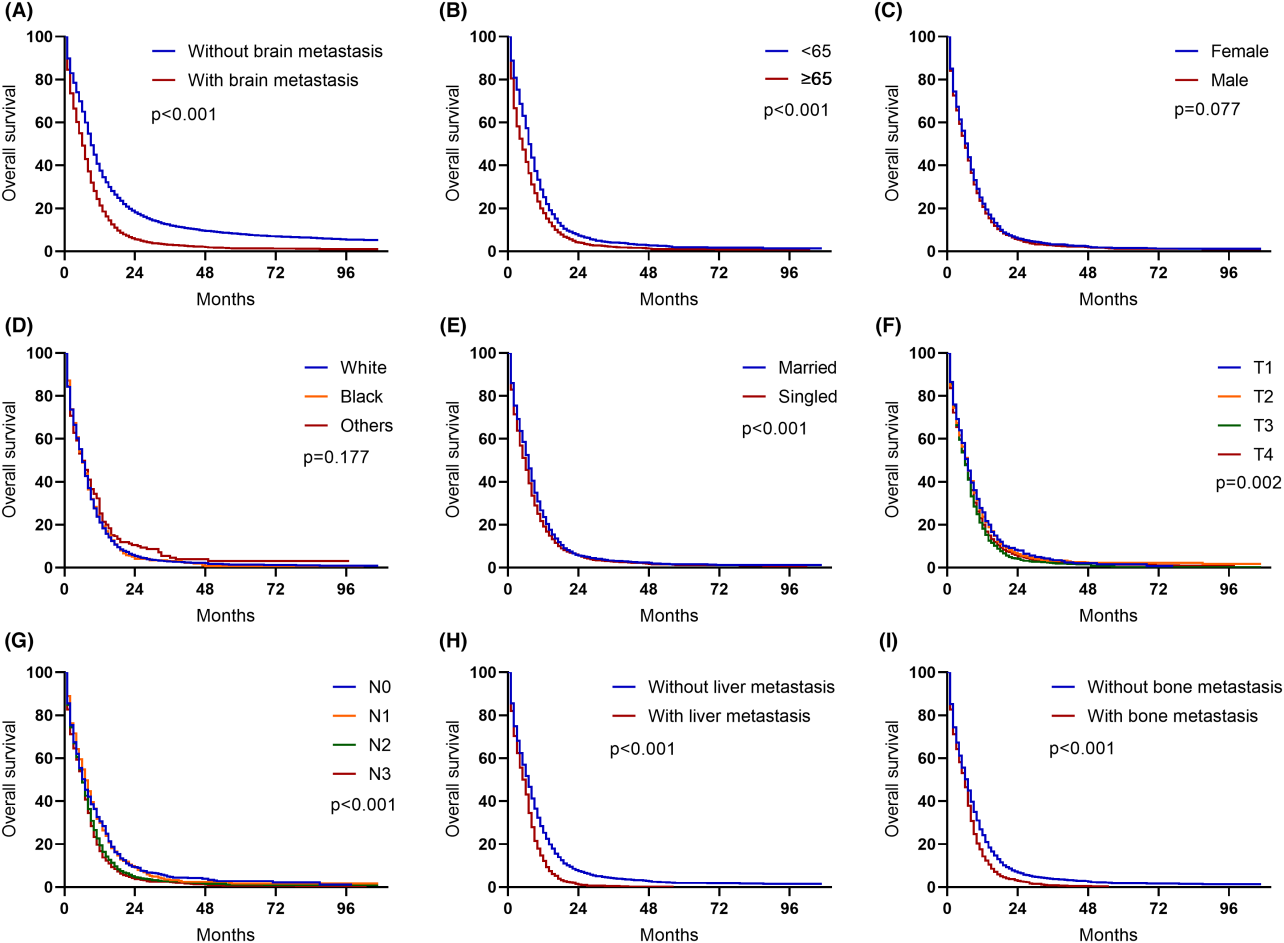
Kaplan–Meier curves of SCLC patients based on the presence of synchronous brain metastases.

The median follow‐up period was 6 months (range, 0–107 months). 85.7% of patients died at the end of the timepoint. The 1‐year and 3‐years cancer‐specific survival rates of patients with SBM were much shorter than those of patients without SBM (19.8% vs. 34.3% and 3.0% vs. 11.9%, respectively, *p* < 0.001). The median survival for the entire cohort was 7 months but the OS of the SBM group was shorter than those of non‐SBM (5 months vs. 8 month, *p* < 0.0001, Figure 2A). Survival estimates of patients with SBM were further stratified and graphically displayed by age (Figure 2B), gender (Figure 2C), race (Figure 2D), marital status (Figure 2E), T stage (Figure 2F), N stage (Figure 2G), liver metastasis (Figure 2H), bone metastasis (Figure 2I). The log‐rank test showed significant difference of the survival curves between age, marital status, T stage, N stage, liver metastases, and bone metastases.

Multivariable Cox regression analysis in Table 2 revealed that the following variables are independently associated with higher risk of mortality: Age ≥ 65 (adjusted HR = 1.164, *p* < 0.025), singled (adjusted HR = 1.095, *p* = 0.012), higher T stage (T3 vs. T1, adjusted HR = 1.265, *p* < 0.001; T4 vs. T1, adjusted HR = 1.192, *p* = 0.002), higher N stage (N2 vs. N0, adjusted HR = 1.347, *p* < 0.001; N3 vs. N0, adjusted HR = 1.452, *p* < 0.001), liver metastases (adjusted HR = 1.415, *p* < 0.001), and bone metastases (adjusted HR = 1.126, *p* = 0.004). Both chemotherapy and radiotherapy are associated with lower risk of mortality (adjusted HR = 0.278, *p* < 0.001; adjusted HR = 0.733, *p* < 0.001). However, tumor location, tumor laterality, number of tumor sites, and lung metastases were not significantly related to the risk of mortality in this test.

**TABLE 2:** Cox regression analysis of OS in SCLC with brain metastases. (Method: Forward LR)

Features	Univariate		Multivariate	
	HR (95%CI)	*p* value	HR (95%CI)	*p* value
Age				
<65	1		1	
≥65	1.359 (1.287–1.436)	<0.001	1.164 (1.086–1.247)	<0.001
Gender				
Male	1		1	
Female	0.951 (0.900–1.004)	0.069	0.916 (0.855–0.982)	0.013
Race				
White	1		1	
Black	0.977 (0.896–1.067)	0.610	0.933 (0.838–1.038)	0.202
Other	0.874 (0.771–0.992)	0.037	0.706 (0.599–0.831)	<0.001
Marital status				
Married	1		1	–
Singled	1.146 (1.084–1.211)	<0.001	1.095 (1.020–1.174)	0.012
Tumor location				
Upper lobe	1		–	–
Middle lobe	0.983 (0.857–1.129)	0.812	–	–
Lower lobe	1.110 (1.037–1.187)	0.003	–	–
Overlapping lesion	0.943 (0.753–1.181)	0.609	–	–
Laterality				
Left	1		–	–
Right	1.009 (0.953–1.067)	0.767	–	–
Both	1.106 (0.983–1.245)	0.095	–	–
Number of primary sites				
1	1		–	–
≥2	1.112 (1.042–1.186)	0.001	–	–
T‐stage				
T1	1		1	
T2	1.040 (0.938–1.153)	0.454	1.075 (0.958–1.208)	0.219
T3	1.194 (1.075–1.326)	0.001	1.265 (1.123–1.425)	<0.001
T4	1.146 (1.041–1.263)	0.006	1.192 (1.066–1.332)	0.002
N‐stage				
N0	1		1	
N1	0.971 (0.860–1.096)	0.632	1.094 (0.940–1.271)	0.245
N2	1.185 (1.091–1.287)	<0.001	1.347 (1.214–1.494)	<0.001
N3	1.244 (1.134–1.366)	<0.001	1.452 (1.292–1.632)	<0.001
Liver metastases				
No	1		1	
Yes	1.488 (1.403–1.578)	<0.001	1.415 (1.306–1.533)	<0.001
Lung metastases				
No	1		–	–
Yes	1.256 (1.171–1.347)	<0.001	–	–
Bone metastases				
No	1		1	
Yes	1.233 (1.160–1.309)	<0.001	1.126 (1.039–1.221)	0.004
Chemotherapy				
No	1		1	
Yes	0.292 (0.274–0.311)	<0.001	0.278 (0.256–0.303)	<0.001
Radiotherapy				
No	1		1	
Yes	0.538 (0.506–0.571)	<0.001	0.733 (0.676–0.795)	<0.001
Surgery				
No	1		–	–
Yes	0.563 (0.414–0.766)	<0.001	–	–

### 3.3 Effects of treatment regimens on OS in patients with SBM

**FIGURE 3:**
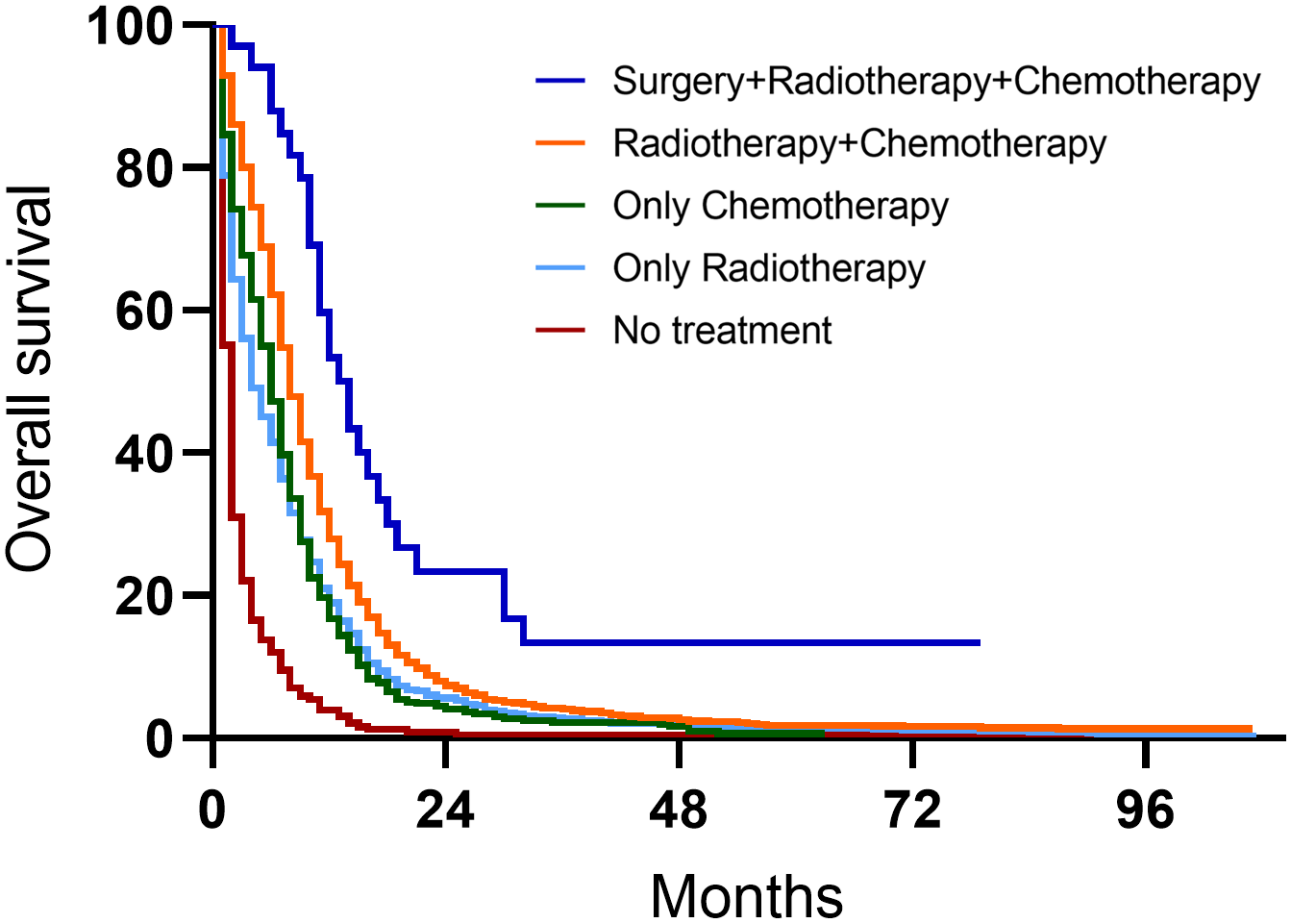
Kaplan–Meier curves of patients who received different treatment regimen.

Table 1 compares the rates of different treatment regimens for SCLC patients with or without SBM. 70.1% of all SCLC patients received chemotherapy and the ratio between the SBM group and the non‐SBM group had no significant difference (*p* = 0.325). 48.9% of all SCLC patients received radiotherapy but the ratio in patients with SBM was significantly higher (*p* < 0.001). The surgery rate in the SBM group was lower than that in the non‐SBM group although the number of patients who accepted surgery was quite small (*p* < 0.001). Figure 3 compares the OS of different therapeutic strategies. The surgery combined with chemotherapy and radiotherapy group exhibited the best prognosis with a median OS of 14 months. The no treatment group unsurprisingly showed the shortest median OS of just 1 month. The median survival time of patients who received combinational treatment of chemotherapy and radiotherapy was 5 months, longer than patients who received chemotherapy or radiotherapy alone. The log‐rank test demonstrated that the results were significantly different.

## 4 DISCUSSION

Small cell lung cancer (SCLC) usually presents highly malignant and metastatic features. It was reported that 70% of SCLC patients have synchronous metastases at the time of initial diagnosis, commonly found in the lymph nodes, brain, liver, and bones.^6^ Interestingly, SCLC takes up less than 20% of lung cancer but accounts for 50% of all lung cancer brain metastases.^7^ The most common sites for tumor brain metastasis are the frontal lobe, the cerebellum, the parietal lobe, and the temporal lobe.^8^ The brain metastases will induce intracranial hypertension, meningeal irritation, motor/sensory disturbances, and epilepsy, leading to poor prognosis and impaired life quality. However, large sample data regarding the demographic features and management of SCLC patients with SBM is limited. In this study, we obtained information of patients of SCLC with or without SBM from the SEER database and explored the relationship between clinical characteristics and the occurrence as well as prognosis of SBM in SCLC, aiming to provide novel evidence‐based support for clinical diagnosis and treatment.

Previous studies have shown that the ratio of SBM in SCLC patients ranges from 15% to 20% and the rate of BM on autopsy in SCLC patients is even higher to 50%.^9^, ^10^ Our study based on the latest data demonstrated that the rate of SCLC patients presenting with SBM at initial diagnosis was 17.2%, higher than the previously reported rate of 16.1% by Reddy et al.^4^ This high rate to some extent indicates the necessity of treatments to prevent brain metastases at the limited stage, such as PCI. In the demographic information part, we found that SCLC patients of age ≥ 65 were less likely to develop SBM but more likely to have shorter survival compared to patients of age < 65. This result was in consistence with a former SEER‐based analysis.^11^ We speculated the reason is perhaps that elder patients tend to show significant symptoms in the early stages and seek medical attention more actively, so they are less likely to develop brain metastases. But once brain metastasis occurred, the physical condition of elder patients is not as good as in younger patients, leading to a poorer prognosis. Table 1 revealed that the proportions of married and singled patients differed between the SBM and non‐SBM groups (*p* < 0.001). Although the logistic regression analysis showed marital status could not be an independent predictor of SBM, single patients tended to have a shorter median OS than married and the difference is statistically significant. The underlying reason might be that single patients lack support both mentally and financially. Our study also showed black people and other races were related to a higher risk of developing SBM, but only other races were related to longer OS. Similar to our study, Reddy et al. found that black patients and American Indian/Alaska Native patients with SCLC were easier to develop SBM than white patients.^4^ However, the results of recent studies often contradict each other and the relationship between race and SBM in SCLC patients is still not fully understood. For example, Wang et al. found that there was a significant difference in OS between white and black SCLC patients with SBM. Goncalves et al. failed to confirm race as a significant predictor of SBM.^11^, ^12^

In terms of tumor specific factors, the multivariate logistic regression analysis revealed that synchronous lung metastases, synchronous bone metastases, and each T stage are independent predictors of SBM, whereas N stage and liver metastases were not. Similar to our results, a retrospective study of Zheng et al. also demonstrated that the T stage was predictive of SBM, whereas nodal metastasis (N stage) was not.^13^ However, the multivariate Cox regression analysis showed that both higher T stages (T3/T4) and N stages (N2/N3) are related to higher mortality. The T stage represents the primary tumor size and invasion ability, and the high T stage means that the tumor is in the advanced stage and more aggressive. Tumors with a higher T stage are prone to distant metastases including the brain, which has been proven by plenty of previous studies.^13^, ^14^ N stage reflects tumor dissemination in lymph nodes. As the CNS lacks the lymphatic system, the only way for SCLC to invade the brain is via the bloodstream and cross the blood–brain barrier (BBB).^7^ Therefore, the nodal invasion may not be associated with the occurrence of brain metastases, but a higher N stage usually indicates that the tumor is in the extensive stage and the prognosis is poor. Interestingly, our study revealed that synchronous lung metastasis is a strong predictor of SBM but not a factor related to patients' survival, which was just opposite to synchronous liver metastases. Although a previous SEER investigation by Ren et al. has demonstrated liver metastasis is the worst prognostic factor in SCLC patients with distant metastasis, the mechanism of this opposite effect is unclear, and more in‐depth studies are needed to confirm this conclusion both biologically and clinically.^15^

Individualized prediction of the prognosis of SCLC patients with SBM is critical to guide clinical decision‐making. Due to the heterogeneity of patients, the traditional TNM staging system was not accurate to predict the prognosis of SCLC with SBM to some extent. According to our result, age, marital status, T stage, N stage, liver metastases, and bone metastases are independently associated with shorter OS and higher risk of mortality. A recent study has established a nomogram and risk classification system to predict the prognosis of patients of SCLC with SBM.^16^ The predictors of this nomogram included sex, age, race, T stage, N stage, and marital status, which are consistent with our results. Our study also revealed that both chemotherapy and radiotherapy are positively associated with better prognosis.

According to the version 1.2020 NCCN guidelines of SCLC, etoposide‐platinum chemotherapy combined with thoracic radiotherapy is still the standard treatment for SCLC. Surgery is only recommended to a small proportion of LS‐SCLC patients.^17^ In this study, most SCLC patients with SBM (85.3%) received chemotherapy, radiotherapy, or chemotherapy combined with radiotherapy and only 0.86% received surgery. Intriguingly, patients who received surgery combined with chemotherapy and radiotherapy exhibited the best prognosis. However, because this part of patients is quite small, the conclusion needs to be further confirmed in large sample studies. Patients who received chemotherapy combined with radiotherapy took a large proportion and showed a better prognosis than both chemotherapy and radiotherapy alone. This is in accordance with clinical practice. According to the decision of a multidisciplinary panel of European experts, chemotherapy alone is recommended as first‐line treatment in asymptomatic SCLC patients with SBM. While for symptomatic patients, WBRT followed by chemotherapy was recommended most.^18^ Although detailed information about treatment cannot be obtained in SEER, our study confirmed the benefits of combination treatment of chemotherapy and radiotherapy for SCLC patients with SBM.

There are several limitations in our study. First, although we selected patients according to a flowchart and excluded patients who did not meet the inclusion criteria, there were some patients with unspecific information on the selected variables. This would influence the accuracy and objectivity of the results. Second, the database lacks information about the details of the treatment. For example, there are usually two kinds of radiotherapies received by patients: Whole‐brain radiotherapy (WBRT) and stereotactic radiosurgery (SRS). Information loss on radiotherapy plans prevented us from comparing the effects of different treatments on prognosis. Third, some other information, such as family history and smoking history, was also missing in SEER database, which may cause bias in the present study. However, our study provides useful information on SCLC with SBM based on this large sample size investigation and the availability of patient demographic information as well as long‐term follow‐up data. Such limitations will not impair the clinical value of our conclusions and augmenting with other data sources will provide more accurate information in the future.

## 5 CONCLUSION

Our study identified the predictive value of clinical characteristics on risk and prognosis of SCLC patients with SBM. Patients with higher T stage, bone metastases, and lung metastases have greater odds of SBM at initial diagnosis. Age ≥ 65, higher T stage (T3/T4), higher N stage (N2/N3), synchronous liver or bone metastases are confirmed to be predictors of poor prognosis of SCLC patients with SBM. Analysis of therapeutic strategies showed that the combination treatment of chemotherapy and radiotherapy benefits patients more than chemotherapy or radiotherapy alone.

## AUTHOR CONTRIBUTIONS

Gang Zhou: Conceptualization, Methodology, Writing the original draft. Zhibo Zheng: Data curation, Visualization, Investigation. Zhiyuan Zhang: Writing and Editing. Yongning Li and Jun Gao: Review of the manuscript. All authors reviewed and approved the final manuscript.

## FUNDING INFORMATION

This study was supported by grants from Tsinghua University‐Peking Union Medical College Hospital Initiative Scientific Research Program (Grant No.2019ZLH206).

## CONFLICT OF INTEREST

The authors have no conflicts of interest to declare.

## ETHICAL APPROVAL AND INFORMED CONSENT

The Institutional Review Board (IRB) of Peking Union Medical College Hospital (PUMCH) has reviewed the above‐named protocol and determined that this study is exempt from full IRB review and informed consent.

## Data Availability

Data sharing is not applicable to this article as no new data were created or analyzed in this study.
